# Online Plain Language Tool and Health Information Quality: A Randomized Clinical Trial

**DOI:** 10.1001/jamanetworkopen.2024.37955

**Published:** 2024-10-08

**Authors:** Julie Ayre, Carissa Bonner, Danielle M. Muscat, Erin Cvejic, Olivia Mac, Dana Mouwad, Heather L. Shepherd, Parisa Aslani, Adam G. Dunn, Kirsten J. McCaffery

**Affiliations:** 1 Sydney Health Literacy Lab, Sydney School of Public Health, Faculty of Medicine and Health, The University of Sydney, Sydney, New South Wales, Australia; 2 Menzies Centre for Health Policy and Economics, Faculty of Medicine and Health, The University of Sydney, Sydney, New South Wales, Australia; 3 Western Sydney Local Health District, Health Literacy Hub, Westmead, New South Wales, Australia; 4 Susan Wakil School of Nursing and Midwifery, Faculty of Medicine and Health, The University of Sydney, Sydney, New South Wales, Australia; 5 Sydney Pharmacy School, Faculty of Medicine and Health, The University of Sydney, Sydney, New South Wales, Australia; 6 Biomedical Informatics and Digital Health, School of Medical Sciences, Faculty of Medicine and Health, The University of Sydney, Sydney, New South Wales, Australia

## Abstract

**Importance:**

Complex and ineffective health communication is a critical source of health inequity and occurs despite repeated policy directives to provide health information that is easy to understand and applies health literacy principles.

**Objective:**

To evaluate the effectiveness of the Sydney Health Literacy Lab Health Literacy Editor, an easy-to-use online plain language tool that supports health information providers to apply health literacy guidelines to written health information.

**Design, Setting, and Participants:**

This randomized clinical trial, conducted online in Australia from May 2023 to February 2024, included a convenience sample of health information providers with no previous experience using the Health Literacy Editor. Analysts were blinded to study group.

**Intervention:**

Participants were randomized 1:1 to the intervention or control group. Participants in the intervention group were provided access to the Health Literacy Editor and a 30-minute online training program prior to editing 3 prespecified health texts. The Health Literacy Editor gives objective, real-time feedback on words and sentences. Control participants revised the texts using their own standard health information development processes.

**Main Outcomes and Measures:**

The preregistered primary outcome was the text school grade reading score (using a validated instrument, the Simple Measure of Gobbledygook). Secondary outcomes were text complexity (percentage of text using complex language), use of passive voice (number of instances), and subjective expert ratings (5-point Likert scale corresponding to items on the Patient Education Materials Assessment Tool).

**Results:**

A total of 211 participants were randomized, with 105 in the intervention group and 106 in the control group. Of 181 participants in the intention-to-treat analysis (mean [SD] age, 41.0 [11.6] years; 154 women [85.1%]), 86 were in the intervention group and 95 in the control group. Texts revised in the intervention group had significantly improved grade reading scores (mean difference [MD], 2.48 grades; 95% CI, 1.84-3.12 grades; *P* < .001; Cohen *d*, 0.99), lower text complexity scores (MD, 6.86; 95% CI, 4.99-8.74; *P* < .001; Cohen *d*, 0.95), and less use of passive voice (MD, 0.95 instances; 95% CI, 0.44-1.47 instances; *P* < .001; Cohen *d*, 0.53) compared with texts revised in the control group in intention-to-treat analyses. Experts rated texts in the intervention group more favorably for word choice and style than those in the control group (MD, 0.44; 95% CI, 0.25-0.63; *P* < .001; Cohen *d*, 0.63), with no loss of meaning or content.

**Conclusions and Relevance:**

In this randomized clinical trial, the Health Literacy Editor helped users simplify health information and apply health literacy guidelines to written text. The findings suggest the tool has high potential to improve development of health information for people who have low health literacy. As an online tool, the Health Literacy Editor is also easy to access and implement at scale.

**Trial Registration:**

ANZCTR Identifier: ACTRN12623000386639

## Key Points

**Question:**

Can an online automated tool, the Health Literacy Editor, help health information providers apply health literacy principles to simplify written health information?

**Findings:**

In this randomized clinical trial of 211 health information providers, people who used the Health Literacy Editor more effectively simplified health information compared with control participants. On average, the intervention group produced texts suitable for a person with significantly fewer years of school education compared with the control group.

**Meaning:**

The findings suggest that the Health Literacy Editor is a scalable tool that can support development of written health information that adheres to plain language principles in clinical and nonclinical settings.

## Teaser

This randomized clinical trial investigates whether an online text editing tool is effective in helping health information providers simplify written health information according to health literacy and plain language guidelines.

## Introduction

National and international policies recognize that health literacy—a person’s capacity to access, understand, and act on health information—is a critical source of inequity in our health systems.^1,2,3^ Low health literacy contributes to higher mortality, morbidity, rates of hospitalization, emergency department visits, and medication errors independently of other social determinants of health, such as age, education, and socioeconomic disadvantage.^4^ A key feature of these policies is the directive to provide health information that all people can easily understand, including people with low health literacy.^5^ There has been a failure to systematically integrate such directives into routine public health and clinical practice despite some of these policies existing for over a decade.^6,7,8,9,10,11^

To support the provision of easy-to-understand health information, there are several freely available, comprehensive guidelines that provide advice about how to apply health literacy and plain language principles to health information.^12,13,14,15,16^ These guidelines recommend evidence-based strategies to improve knowledge and recall of health information, such as putting essential information first, using simple language, and minimizing medical jargon.^17,18^ However, accompanying systems, training, and tools are needed to drive meaningful change in health literacy practices within an organization.^19,20^

Online tools are well placed to help improve the application of health literacy guidelines because of their capacity to provide specific, immediate, and actionable feedback on written text.^21,22,23^ These tools typically identify difficult words, sentences, and grammatical structures and sometimes integrate technologies, such as natural language processing and artificial intelligence.^24,25,26,27,28,29,30^ However, few have been specifically designed for health contexts,^28,29,30^ and only 2 have been formally evaluated; both evaluations were limited by small sample size and pre-post study design.^29,30^ Though these tools hold promise, it is unclear how effectively health information providers incorporate the tool’s feedback when revising and simplifying health information.

To address this research gap, the current study aimed to evaluate whether using the Sydney Health Literacy Lab (SHeLL) Health Literacy Editor (hereafter, Health Literacy Editor) can support health information providers to effectively apply health literacy guidelines to written health information. The Health Literacy Editor is a new online tool that provides objective and immediate assessment of written health information across a range of factors, including feedback on school grade reading levels, complex language, passive voice, text structure, lexical density and diversity, and person-centered language.^31^ The tool guides users in real time, providing simpler or more familiar alternatives for medical and other words, and demonstrates to the user how small changes can incrementally increase use of plain language in written health information.

## Methods

### Study Design

This randomized clinical trial used a 2-arm, parallel-group study design with participants randomly assigned to the intervention or control group (Figure and trial protocol in Supplement 1). This trial was prospectively registered with the Australian New Zealand Clinical Trials Registry (ACTRN12623000386639) and approved by The University of Sydney Human Research Ethics Committee. Completion of the online survey indicated informed consent. Participants were recruited between May 31 and November 27, 2023, with follow-up completed February 26, 2024. Participants received an AU$50 (US$34) gift card to thank them for their time. Reporting of results adhered to the Consolidated Standards of Reporting Trials (CONSORT) reporting guideline.^32^

**Figure.**
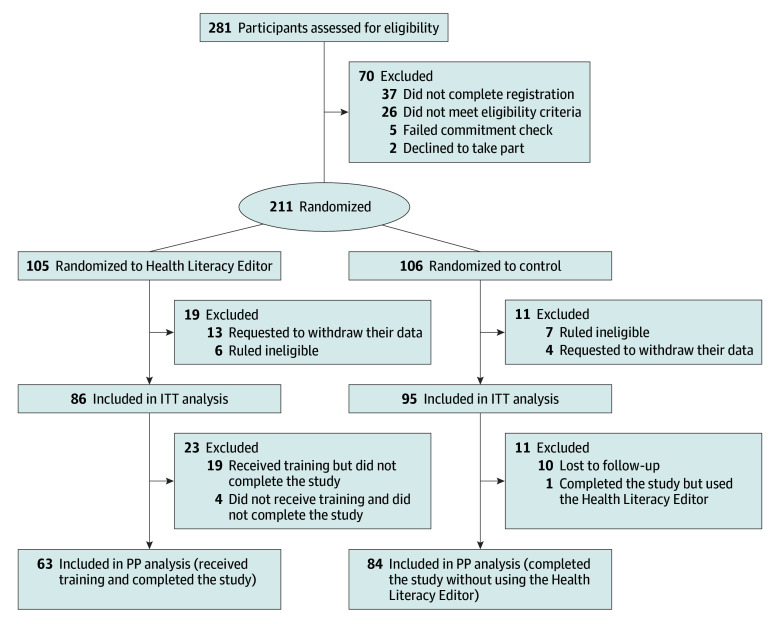
CONSORT Flow Diagram Participants were ruled ineligible if they entered multiple times with the same email address. ITT indicates intention to treat; PP, per protocol.

### Participants

Eligible participants were adults in Australia whose work involved developing health information. Students in medicine, allied health, and health sciences were also eligible. Participants had to positively affirm their commitment to the trial (“Do you commit to providing thoughtful answers in this study?”). Participants were not eligible to take part if they had previous experience using the Health Literacy Editor. Participants were recruited online through health networks, newsletters, and social media.

### Study Groups

#### Health Literacy Editor Group

The Health Literacy Editor is a browser-based software application that gives objective, real-time feedback on the complexity of health information. The tool comprises 6 assessments: readability as measured by the Simple Measure of Gobbledygook (SMOG) formula,^33^ complex language, passive voice, text structure, lexical density and diversity, and person-centered language. These are each presented as global scores that update as the user edits the text. The tool also provides more specific feedback flagged in the text through highlighting individual words and sentences that are relevant to each assessment. For example, the tool flags long words that will contribute to a poorer readability score. Full details of the development are published elsewhere.^31^ User testing with health staff has helped improve the quality of training, instructions, and feedback that the tool provides.^34^ Participants randomized to this group attended a 30-minute online training session in which they learned how to use the tool. Three training resources were also embedded within the tool: a help page that contained instructions, video tutorials, and worked examples; a quiz to check understanding of key concepts; and a 2-page PDF introducing key concepts. After completing training, participants were instructed to use the Health Literacy Editor to help them revise 3 health texts.

#### Control Group

Participants randomized to the control group were asked to use their usual processes to revise the 3 health texts. No further training was provided.

### Procedure

Participants provided demographics, details about their professional or student role, and their experience developing health information. They were then randomized 1:1 to the intervention or control group using the Qualtrics survey platform Mersenne Twister algorithm. Participants were emailed a link to a second survey that asked them to revise 3 health texts, each approximately 200 words and written at a grade 14 reading level, on the topics of dementia, cancer, and sciatica (eAppendix 1 in Supplement 2). Three topics relating to different commonly occurring health conditions were selected to mitigate bias attributable to topic area expertise. Selection of the texts ensured that there were enough long words and sentences, complex language, and instances of passive voice that participants could demonstrate their ability to simplify the text according to health literacy guidelines.

Instructions for both study groups asked participants to revise each text to make it easy for most people to understand, aim for a grade 8 to 10 reading level, and retain any key messages within the text. The reading level range was selected to reduce participant burden and ensure the revision task was feasible. To aid revision, a brief description of the purpose for each text was also provided. After revising the texts, participants completed items about self-reported estimates of time taken to revise the text. Participants in the intervention group reported the features that they used and the tool’s usability and acceptability.

### Outcomes

#### Primary Outcome

The primary outcome was grade reading score as measured by SMOG.^35^ The grade reading score provides an estimate of text difficulty that corresponds to the US grade reading level required to understand the meaning of a text. In Australia, health literacy guidelines recommend that information is written at a grade 8 reading level or lower.^36^

The SMOG formula is widely used in health literacy research^33^ and calculates grade reading score based on the number of long words (defined as 3 syllables or more) and the total number of sentences.^35^ The resulting score corresponds to the grade reading level at which a person would be expected to correctly answer 100% of multiple-choice comprehension questions. SMOG is the only readability formula that uses 100% comprehension for calibration. Compared with other formulas, the SMOG score is more consistent across random samples from a given text and less sensitive to differences in formatting.^33^ The SMOG score produced by the Health Literacy Editor is closer to the reference standard (ie, hand-calculated scores) than SMOG scores produced by other software.^37^

#### Secondary Outcomes

We assessed several secondary outcomes. These included objective text complexity and objective passive voice, as assessed by the Health Literacy Editor, as well as subjective expert ratings, time to complete text revisions, intervention acceptability, and intervention engagement. The Health Literacy Editor's complex language and passive voice assessments were custom built, as there were no other existing validated formulas or programming available that we could incorporate into the tool.

Using simple everyday language is a key health literacy recommendation to improve the quality of health information.^12^ The complex language assessment in the Health Literacy Editor reports the proportion (percentage) of words that are uncommon in English, acronyms, or words with a suggested alternative in the tool’s thesaurus.^37^ It draws from several resources to identify complex language, including a database comprising more than 270 million words from diverse English-language sources to identify words that are uncommon in English.^38^ Objective text complexity was assessed using the Health Literacy Editor’s complex language score.

Using active voice is a key recommendation to improve how easy health information is to understand.^12^ Objective passive voice was assessed using the Health Literacy Editor. Its passive-voice assessment uses natural language processing to identify patterns of the verb *to be* (eg, *is*, *were*) and a past participle (eg, *delivered*, *given*) that indicate passive voice.^37^

The Patient Education Materials Assessment Tool (PEMAT) is a widely used and validated health literacy tool that asks the assessor to subjectively rate whether printed health materials adhere to 24 items.^12^ These scores provide an estimate of understandability (ie, how easy a material is to understand) and actionability (ie, how easy a material is to act on). Topics include content, word choice and style, use of numbers, organization, layout and design, and use of visual aids. In this study, 2 coinvestigators with expertise in health literacy (J.A., O.M.) assessed each revised text according to the PEMAT topics content and word choice and style. The content topic relates to a clear purpose and absence of distracting information. Word choice and style refers to use of common, everyday language, minimal and defined medical terms, and use of the active voice where possible. A third rating, retained meaning, reflected whether key messages were retained in the revised texts. This was added to ensure texts were not simplified by removing content. Experts were masked to study group. The subjective expert rating scores represent average ratings on a 5-point Likert scale (strongly disagree [1] to strongly agree [5]), and each participant’s expert rating score reflected the mean of the 6 scores (3 texts, each rated by 2 experts).

Participants in both study groups were asked to estimate the number of minutes to complete all 3 revision tasks. Time to complete text revisions was recorded.

Participants in the intervention group provided acceptability ratings via 2 validated instruments. The System Usability Scale^39,40^ produces a score from 0 (low) to 100 (high). A score of 70 is considered acceptable and a score of 90 or higher is considered superior.^40^ The Technology Acceptance Model^41^ comprises 2 subscales, including perceived usefulness and perceived ease of use, with scores ranging from 1 (low) to 5 (high). Scores are predictive of current and future use of a product.^41^

Participants in the intervention group were also asked with which Health Literacy Editor features they engaged when revising the text. Participants who reported using at least 2 of 3 key features described in training (readability, complex language, and passive voice) were assessed as having adequate engagement.

### Patient and Public Involvement

A community member was involved in discussions about study design. Methods and outcome measures related to community ratings of revised texts incorporated the community member’s feedback. This subsequent component of the project is not reported in this article and will be reported separately. Several health services staff and university research staff members helped pilot and improve the training materials and ensured that the text revision task was feasible without placing undue burden on participants.

### Sample Size

A sample size estimate of 120 (60 participants per group) was calculated to have 90% power at α = .05 to detect a moderate effect size (Cohen *f*, 0.30) in the main outcome (grade reading score). An additional buffer allowed for up to 33% dropout before the text revision task was completed, for a total of 180 participants needed. Sample size was adjusted during recruitment to account for a larger-than-expected noncompletion rate (n = 211).

### Statistical Analysis

Univariable (simple linear) regression models analyzed differences for resources developed using the Health Literacy Editor (averaged across the 3 texts) and those developed by participants in the control condition using 2-sided hypothesis tests. For highly skewed secondary outcomes, nonparametric (Mann-Whitney *U*) tests were used. Histograms depicting frequency distributions are available in eAppendix 2 in Supplement 2.

Intention-to-treat (ITT) and per-protocol (PP) analyses are presented. For ITT, the scores of the original texts were retained as the scores for revised texts for participants who did not complete the revision task. That is, we assumed no changes were made to the text. The PP analysis included only participants who submitted revised versions of the texts (for participants in the intervention group, this included attendance at training). One participant in the control group who used the Health Literacy Editor was included in the ITT analysis and excluded from the PP analysis. Descriptive statistics summarized information about usability, acceptability, and engagement (intervention group only). For all subjective ratings, assessors were unaware of the study group.

Analyses were performed using SPSS, version 26 (IBM Corp). The threshold for significance of the primary outcome was *P* < .05, and all hypothesis tests were 2-sided. The same significance threshold was used for analyses of secondary outcomes and can be interpreted as exploratory.

## Results

### Sample Characteristics

A total of 211 participants were randomized, with 105 in the intervention group and 106 in the control group. Among 181 participants included in the ITT analysis (86 in the intervention group and 95 in the control group), the mean (SD) age was 41.0 (11.6) years; 24 (13.3%) identified as men, 154 (85.1%) as women, and 3 (1.7%) as nonbinary or other gender; and most reported working in health services (113 [62.4%]) and/or government organizations (117 [64.6%]) (Table 1). Characteristics appeared comparable for the 147 participants in the PP sample (63 in the intervention group and 84 in the control group) (eTable 1 in Supplement 2).

**Table 1.** Participant Characteristics by Study Group in the ITT Analysis

Variable	Participants, No. (%) (N = 181)	
	Health Literacy Editor (n = 86)	Control (n = 95)
Age, mean (SD), y	41.0 (11.4)	40.6 (11.8)
Gender		
Men	11 (12.8)	13 (13.7)
Women	74 (86.0)	80 (84.2)
Nonbinary or third gender	1 (1.2)	2 (2.1)
Role^a^		
Staff only	71 (82.6)	77 (81.1)
Student only	2 (2.3)	2 (2.1)
Student and staff	13 (15.1)	16 (16.8)
Engagement, staff only^a^		
Consumer advocacy group	0	2 (2.1)
Government	56 (65.1)	61 (64.2)
Health services	54 (62.8)	59 (62.1)
Industry	2 (2.3)	7 (7.4)
Not-for-profit or charity	3 (3.5)	4 (4.2)
University or tertiary education	7 (8.1)	10 (10.5)
How often do you develop or revise written health information for patients or the community?		
Daily	16 (18.6)	19 (20.0)
Weekly	15 (17.4)	13 (13.7)
Monthly	15 (17.4)	17 (17.9)
A few times a year	26 (30.2)	29 (30.5)
Once	2 (2.3)	1 (1.1)
Never	10 (11.6)	14 (14.7)

Abbreviation: ITT, intention to treat.

^a^ Multiple roles and engagements could be selected.

The number and flow of participants at each stage is shown in the Figure. Most participants completed follow-up (147 [81.2%]), though this rate was lower for participants in the intervention group compared with the control group (63 [73.3%] and 84 [88.4%], respectively). Tools that participants in the control group reported using are shown in eTable 2 in Supplement 2.

### Evaluation of Revised Texts

#### Primary Outcome

Compared with texts revised by participants in the control group, the texts revised by those in the intervention group had significantly improved grade reading level (mean difference [MD], 2.48 grades; 95% CI, 1.84-3.12 grades; *P* < .001; Cohen *d*, 0.99) (Table 2 and eTable 3 in Supplement 2). Magnitude of effects was larger in the PP analysis, with participants in the intervention group reducing the grade reading level by more grades compared with those in the control group (MD, 3.79 grades; 95% CI, 3.29-4.28 grades; *P* < .001; Cohen *d*, 1.58).

**Table 2.** Participant Scores for Revised Materials by Study Group

Variable	Original text score	Participant score (N = 181)^a^			
		Intention to treat		Per protocol	
		Intervention (n = 86)	Control (n = 95)	Intervention (n = 63)	Control (n = 84)
Objective assessments					
Words, No.	195.00	188.75 (23.91)	177.79 (32.4)	185.46 (26.09)	176.21 (33.57)
Grade reading score	13.97	9.98 (2.68)	12.46 (1.59)	8.52 (1.37)	12.31 (1.60)
Text complexity, %	25.87	15.19 (7.40)	22.05 (5.31)	11.32 (4.25)	21.67 (5.49)
Instances of passive voice, No.	5.00	2.95 (1.75)	3.90 (1.75)	2.18 (1.40)	3.82 (1.80)
Subjective expert rating^b^					
Content, median (IQR)	5.0	5.00 (5.00-5.00)	5.00 (5.00-5.00)	5.00 (5.00-5.00)	5.00 (5.00-5.00)
Word choice and style	2.7	3.70 (0.74)	3.26 (0.58)	4.07 (0.45)	3.32 (0.58)
Meaning retention, median (IQR)	5.0	5.00 (4.67-5.00)	5.00 (4.83-5.00)	4.83 (4.67-5.00)	5.00 (4.71-5.00)

^a^ Data are presented as mean (SD) unless otherwise indicated.

^b^ Five-point Likert scale, with 1 indicating strongly disagree and 5, strongly agree.

#### Secondary Outcomes

The same pattern was observed for secondary outcomes, with texts revised by those in the intervention group showing lower text complexity score (MD, 6.86; 95% CI, 4.99-8.74; *P* < .001; Cohen *d*, 0.95) and fewer instances of passive voice (MD, 0.95 instances; 95% CI, 0.44-1.47 instances; *P* < .001; Cohen *d*, 0.53) (Table 2 and eTable 3 in Supplement 2). Expert ratings for word choice and style (common everyday language, minimal and defined medical terms, and active voice) were higher in the intervention group compared with the control group (MD, 0.44; 95% CI, 0.25-0.63; *P* < .001; Cohen *d*, 0.63) (Table 2). Magnitude of effects for secondary outcomes were larger in the PP analysis, including across both objective and subjective expert ratings. Ratings for content (clear purpose and absence of distracting content) and retaining meaning were high and did not differ significantly between the 2 groups (eTable 4 in Supplement 2).

#### Acceptability and Engagement

On average, participants rated the Health Literacy Editor as an acceptable product that was useful and easy to use (Table 3). Participants using the Health Literacy Editor reported spending a mean (SD) of 65.40 (33.02) minutes revising the 3 texts compared with an estimated mean (SD) of 30.13 (18.28) minutes for the control group. Almost all participants in the intervention group in the PP analysis (59 of 63 [93.7%]) reported using all 3 of the Health Literacy Editor key assessments: readability, complex language, and passive voice.

**Table 3.** Acceptability and Engagement With the Health Literacy Editor Among 63 Participants in the Intervention Group in the Per-Protocol Analysis

Characteristic	Value
System Usability Scale score, mean (SD)^a^	70.99 (13.69)
Technology Acceptance Model, mean (SD)	
Perceived usefulness score^b^	3.76 (0.71)
Perceived ease of use score^b^	4.03 (0.59)
Time to complete revisions, min	65.40 (33.02)
Self-reported Health Literacy Editor features used to revise the text, No. (%)	
Readability	62 (98.4)
Complex language	63 (100)
Passive voice	59 (93.7)
All 3 features	59 (93.7)

^a^ Score range of 0 to 100, with 70 considered acceptable and 90 or higher, superior.

^b^ Score range of 1 (low) to 5 (high).

## Discussion

This randomized clinical trial found that health information that was revised using the Health Literacy Editor more closely aligned with health literacy and plain language guidelines compared with texts revised according to participants’ standard processes. The texts revised using the Health Literacy Editor had a lower grade reading score and used less complex language and passive voice, showing greater potential to meet the health literacy needs of the population, including people who are older, who have had less opportunity for education, and who speak English as a second language. Subjective ratings from health literacy experts provided further evidence that these revised texts were clear and retained the original meaning. Though participants who used the Health Literacy Editor took longer to revise the texts, we believe this time investment is reasonable given the magnitude of effects, the likelihood that participants may become faster with repeated use of the tool, and the tool’s strong capability to support scalable and easily accessible health literacy training.

These findings highlight that innovative new tools can meaningfully contribute to bridging the well-documented gap between health literacy policy and practice.^1,2,6,7,8,9^ To date, several promising tools have been developed,^24,25,26,27,28,29,30^ with limited evaluation of their effectiveness.^29,30^ To our knowledge, this is the first randomized clinical trial to show that health literacy software providing objective, real-time, and fine-grained feedback on words and sentences was effective in supporting health information providers to develop plain language written materials. Coupled with sound user-acceptability ratings, further work is now needed to explore how tools such as the Health Literacy Editor can be implemented at scale within an organization and to evaluate its effects on patient outcomes.

Online tools are well placed to support consistent and scaled uptake of health literacy guidelines by a workforce that may have received little training. This is important given that developing health information can be an intermittent activity for clinicians and health staff, particularly when roles are transient or project based. Online tools and training have the advantage of being easily accessed online without geographic or time constraints. Developing health texts will always need human oversight and expertise. We envisage that health information providers would use the Health Literacy Editor in combination with other tools and strategies while maintaining existing quality and safety processes for clinical oversight. These caveats are likely to continue to apply even with advances in artificial intelligence that may provide additional practical benefit by quickly and coherently simplifying health information.^42,43^ Further research into the potential of artificial intelligence for developing simple health information is warranted.

### Strengths and Limitations

In addition to the randomized clinical trial design, several other aspects further strengthen the study findings. For example, though readability is an appropriate primary outcome measure, it has been criticized as a narrow indicator of plain language.^44^ This study included a variety of objective and subjective assessments of plain language (complex language and passive voice) and was able to show consistent patterns across a wide range of outcomes. Study findings were also strengthened by asking participants to submit 3 revised texts on different health topics. This reduced the likelihood that content area expertise would influence results.

There are also some limitations to this study. Several participants completed intervention training but did not submit revised texts. It is possible that some were overwhelmed by the training or did not see value in the tool if they were already confident in their skills. This may also be a product of the time-intensive nature of the task. Qualitative and codesign research may further improve training and help set appropriate expectations for using the tool. It is unclear whether results generalize to health information developers in nongovernment sectors given the low number of participants from industry, consumer advocacy groups, and tertiary institutions. Also, although the health literacy experts were blinded to intervention group, it is possible that they were able to anticipate the kinds of changes that the Health Literacy Editor would suggest. Future studies could include masking checks or involve experts who are explicitly unfamiliar with the tool. In addition, it is unclear whether improved uptake of plain language will translate to improved perceptions of the health information by consumers and patients. Further work is under way to explore whether consumers prefer and can more easily understand texts developed using the Health Literacy Editor. This work may also help understanding of the relative importance of each objective assessment.

## Conclusions

In this randomized clinical trial, the Health Literacy Editor supported users in applying health literacy and plain language strategies to written text while retaining key content and meaning. New technologies may make an important practical contribution to achieving the goals set out by health literacy policy for clear health communication, improved health equity, and better health outcomes. These tools have potential to improve health outcomes for people with lower health literacy.

## Notes

Supplement 1.
Trial Protocol

Supplement 2. **eAppendix 1.** Materials for Text Revision Task **eAppendix 2.** Frequency Histograms for Outcome Variables **eTable 1.** Participant Characteristics by Intervention Group, ITT and PP Analysis Samples **eTable 2.** Reported Resources or Tools Used by the Control Group, PP Sample Only **eTable 3.** Effect Size Estimates **eTable 4.** Descriptive Statistics for Outcome Variables Undergoing Nonparametric Tests

Supplement 3.
Data Sharing Statement
