# Supplementary material for: Online Plain Language Tool and Health Information Quality: A Randomized Clinical Trial
Source: JAMA Netw Open. 2024 Oct 8;7(10):e2437955. doi: 10.1001/jamanetworkopen.2024.37955 (PMC11581667; doi:10.1001/jamanetworkopen.2024.37955)
Supplement: Supplement 1. — Trial Protocol [file jamanetwopen-e2437955-s001.pdf]

eAppendix 1: Study protocol

CheckMyText! Evaluation of the SHeLL Health Literacy Editor

Background

Low health literacy affects more than half (60%) of the Australian population[1] and is associated with higher mortality, morbidity, rates of hospitalisation and emergency department visits, and medication errors.[2] National and international policies recognise health literacy as a critical source of health inequality that disproportionately affects those who are older, less educated, and people from culturally diverse communities (2020 National Health Reform Agreement; WHO Sustainable Development Goals).

A key directive of these policies is to provide health information that all people can easily understand including those with low health literacy. Despite being a relatively simple, low-cost strategy to address health literacy, this directive has not been integrated into routine public health and clinical practice. Research estimates less than 1% of online Australian health information is written simply enough for most people to understand.[3]

The current evidence-base for interventions that produce easy-to-read health information at scale is insufficient. Several health literacy guidelines advise how to structure, write, and visually present health information.[4-6] However, except for school grade reading levels, there are no other tools that objectively assess whether individual words or sentences may be hard to understand. As a result, applying health literacy principles to written text is often time-consuming and highly subjective.

The Sydney Health Literacy Lab (SHeLL) Health Literacy Editor is a new tool developed to address this issue. It provides objective assessment of written health information across a range of factors. These include feedback on school grade reading levels, medical jargon, and complex grammatical structures such as the passive voice. The Editor also guides users in real-time, providing suggested alternatives and demonstrating how small changes can incrementally improve the text. The Editor has undergone extensive user testing with health staff to refine the usability of its features and improve acceptability (manuscript currently under review). However, though the Editor assesses wide-ranging factors that influence text complexity, it is unknown whether health information providers can easily act on this feedback to revise and simplify health information.

<u>Aim:</u>

This study aims to evaluate whether health information developers produce higher quality written health information (i.e. easier to understand) using the Health Literacy Editor compared to usual processes.

Part 1: Randomised-controlled trial and process evaluation (health information developers)

Methods

Study design

This is a two-arm randomised-controlled trial comparing the effect of the intervention (Sydney Health Literacy Lab (SHeLL) Health Literacy Editor) and control (usual processes)

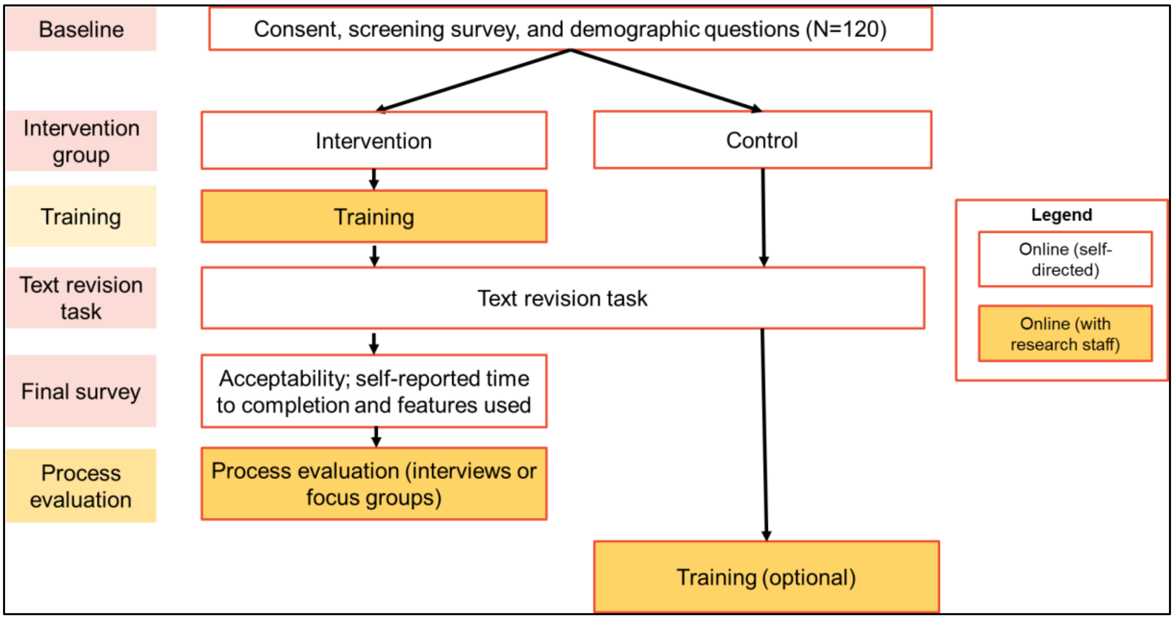

Figure 1. Survey flow

Intervention groups

Intervention (SHeLL Health Literacy Editor): Participants will have access to the Editor (https://shell.techlab.works/) and self-directed training materials. The Editor is an online browser-based tool that helps users develop text-based health education materials that adhere to health literacy guidelines. The Editor comprises six assessments: readability, complex language, passive voice, text structure, lexical density and diversity, and person-centred language. These are each presented as global scores, with additional, more specific feedback flagged in the text itself. Several training resources embedded within the intervention include:

1. **Help page:** The Editor's help page containing instructions, video tutorials and worked examples
2. **Self-check tool:** 5 questions to check understanding of key concepts (available from the help page)
3. **Quick start guide:** A 2-page PDF introducing key concepts (available from the help page)

Participants in this group will also be invited to take part in a 30-minute Zoom call. Participants will be introduced to the tool and any relevant training resources, with opportunity to ask questions. If participants are not able to make the Zoom meeting they will have access to a recording of the same content. Zoom attendance and viewing of the recorded training will be monitored for adherence.

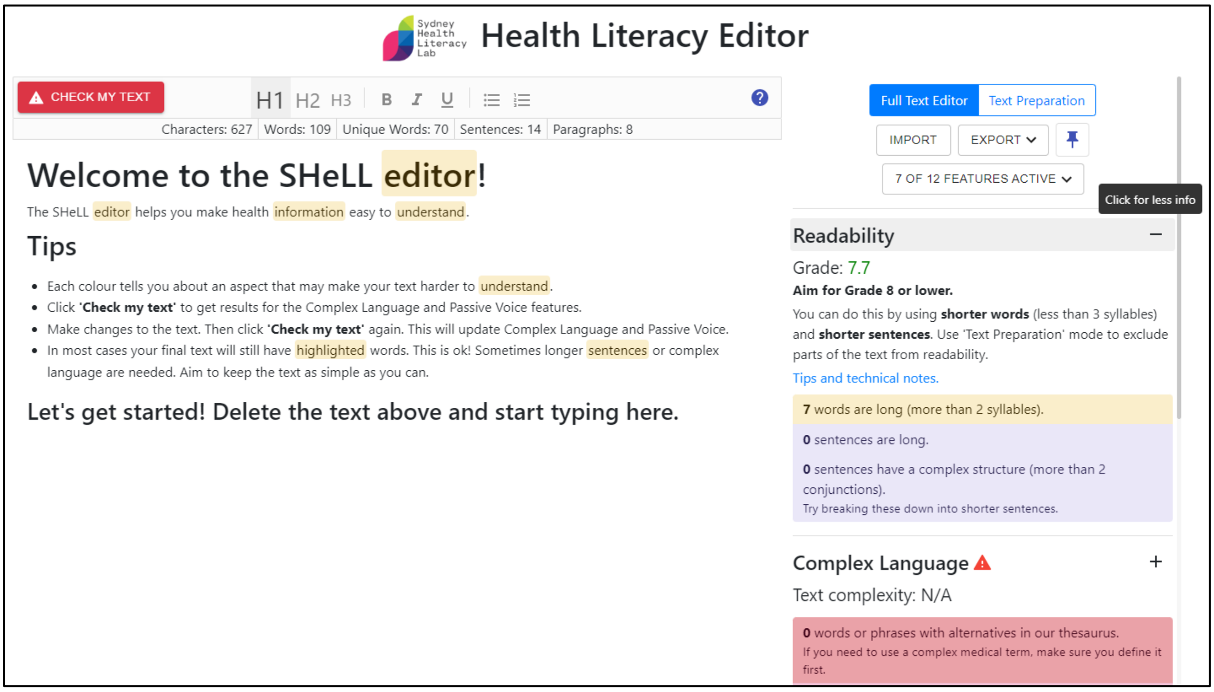

Figure 2. Screenshot of the SHeLL Health Literacy Editor.

Control: Participants will be asked to use their usual processes to revise the text. No training is provided prior to the task revision task. Participants will have the option to take part in SHeLL Health Literacy Editor training *after* completing the revision task (i.e. after completing their participation in the trial).

Participants and recruitment

Participants: Eligible participants are Australian people whose work or study involves developing health information. This can include, for example, health information providers, health services staff, and clinicians. Students in medicine, allied health and health sciences are also eligible to take part. Participants are not eligible to take part if they have previous experience using the Health Literacy Editor.

Participants will be recruited through the investigators' existing networks such as the Health Literacy Hub network which comprises >1000 members across Australia. Information about the study will be circulated via communication platforms such as newsletters and social media.

Participants who complete the text revision task will be provided with a $50 gift card. Participants who take part in the focus groups will receive an additional $50 gift card.

Consent: Participants will be given the participant information statement and can indicate their consent using an online survey (Qualtrics platform). All participants will be asked to provide an email address or phone number by which study researchers may contact them with invitations to the training components, the text revision task and if selected, the interviews. This email address will also be used to remind participants to complete the revision task (see below for more detail). At the end of the trial task, participants in the Health Literacy Editor group can also indicate their consent to take part in the online focus-groups.

Procedure

Survey 1: Following the consent process, the survey will ask for demographic and professional information (approx. time 5 minutes).

Participants will then be randomised to intervention group. Participants allocated to use the intervention will complete training via Zoom (see above, 30 minutes).

Survey 2 (revision task and process evaluation): Participants will then be asked to revise 3 texts (total approx. 600 words, example texts are provided in the attachment 'survey for health information providers.' Texts may be adjusted after piloting to ensure the task is manageable for participants). Participants will be provided with instructions to revise the text so that it is easier to understand. To aid revision, a brief description of the target audience and purpose for each text is also provided. Estimated time to complete the task is 40 minutes. After revising the texts, participants will be asked to complete survey items about self-reported estimates of time taken to revise the text, usability, acceptability, and engagement/fidelity.

Reminder emails: If the revision task is not completed, two reminders will be sent, at 1 week and 2 weeks following from training (intervention group) or consent (control group).

After completion of survey 2, participants will be emailed a $50 gift card to thank them for their time completing the trial.

Measures

Primary outcome: Grade readings score (Simple Measure of Gobbledygook). The Editor uses the SMOG readability formula [8], which is considered a more reliable, robust, and conservative estimate of grade reading score compared to other readability formulas [9-11]. The Editor's automated SMOG scores are more accurate than those of other online SMOG calculators [12]. In Australia, health literacy guidelines recommend that information is written at a grade 8 reading level or lower [13].

Secondary outcomes:

1) **Text accuracy / retention of meaning**: Two researchers will assess each text for retention of key messages (determined a-priori), and identify any additional or incorrect information.
2) **Text Complexity:** The Editor's text complexity score reports the proportion (as a %) of words in the text being assessed that are flagged by the program as 'complex.' This includes acronyms, any words for which a simpler alternative has been identified, based on public health and medical thesauruses, and any words that are flagged as 'uncommon' in English, according to a database of more than 270 million words.
3) **Passive voice:** The Editor's passive voice score indicates the number of passive voice in the text.
4) **Expert PEMAT assessment**: Two co-investigators will assess each revised text, masked to intervention group. Relevant PEMAT items are shown in the table below A third item, retained meaning, was also added.

PEMAT Item

**Content**	
	Makes its purpose completely evident
	No distracting information or content
**Word Choice and Style**	
	Common, everyday language
	Medical terms are defined and used only to familiarise readers
	Active voice

5) **Consumer assessments of information quality: see Part 2 below**
6) Self-reported time to complete the task

Process evaluation

Participants allocated to the intervention (Health Literacy Editor) group will be asked to complete survey items on:

1) **Usability**: assessed by the System Usability Scale [15, 16], and The System Usability Scale produces a score from 0 (low) to 100 (high). A score of 70 is considered 'passable,' and a score of 90 or more is considered indicative of a 'truly superior product' [16].
2) **Acceptability**: the Technology Acceptance Model (comprises two subscales: perceived usefulness and perceived ease of use) [17]. Each subscale produces a score ranging from 1 (low) to 7 (high). Scores are predictive of current and future use of a product [17].
3) **Engagement and fidelity:** Self-reported Health Literacy Editor training resources accessed and features within the tool used when revising the text

Participants in both conditions will be asked to briefly describe any other tools they used to revise the text e.g. Microsoft Word, Thesaurus.com.

Focus groups: A subset of participants in the intervention arm (n=25) will be invited to take part in focus groups to provide further feedback on the Editor and to help identify potential implementation issues. Participants randomised to the Health Literacy Editor can indicate their interest in the focus groups after submitting their revised texts. Participants invited to the focus groups will be contacted via their nominated phone number or email address (see attachment), with one reminder phone call and email provided if needed.

Semi-structured focus group questions will elicit detailed data about components shown in Box 1. We anticipate 4 groups of 4-8 participants each (total approx 20-30 participants) to elicit sufficiently rich data. We will purposively sample participants to ensure there is a range of people with different characteristics e.g. gender, age, experience in varying sectors/professions and varying confidence in producing plain language written health materials.

Box 1. Topic guide for semi-structured focus groups

1. General impressions/feedback on the Health

Literacy Editor

2. Discussion of training resources
3. Reflections on the revision task, including aspects that were easy and challenging
4. Incorporating the Health Literacy Editor into workflows and business as usual
5. Potential barriers and facilitators to embedding the Editor into workflow

After completion of the focus groups participants will be emailed a gift voucher ($50).

Analysis

Sample size: Sample size estimate of 120 (60 participants per group) is based on 90% power at α = 0.05 to detect a moderate effect size (Cohen's f = 0.30) in the main outcome (grade reading score). An additional buffer (total 144) will allow for 20% drop-out before the text revision task is completed.

Statistical analysis: Univariable regression models will be performed to analyse differences in information quality for resources developed using the Health Literacy Editor (intervention) and control (Grade Reading Score as primary outcome). Participants in the control group who state that they have used the Heath Literacy Editor to revise the texts will be excluded from the analysis.

Descriptive statistics will be used to analyse usability, acceptability and fidelity (intervention group only).

Qualitative analysis: Focus-groups will be audio-recorded and transcribed, then analysed using thematic analysis. We anticipate a sample size of approximately 25-30, as this is a typical number included for qualitative research.

Part 2: Consumer assessments of revised materials

Study design

This component will provide the secondary outcome measure for part 1 (the consumer assessment of revised texts). Data will be collected via survey.

Participants and recruitment

A subsample of the revised texts will be assessed by two consumers requiring a total 150 consumers. Participants will be recruited via community events and on-site at TAFE NSW venues. Members of the study team will explain the study and the health topics that participants will be asked to read about. Interested participants will then be directed to the landing page of an online survey where they can read the PIS and give informed consent before participating in the study. To be eligible, participants must be adults (18 years and over) with less than a university level of education, or may have a university level of education if this was obtained overseas and English is not their main language. Each participant will receive a $25 gift voucher to thank them for their time.

Survey

A Qualtrics survey will collect demographic data on e.g. age, gender, education, cultural diversity and health literacy (see survey attachment).

Each consumer will assess two sets of paired texts (one original text, one revised by a single participant from part 1). This assessment will be completed using selected adapted items from the 'information and presentation' subscale of the e-Health Impact Questionnaire -Part 2[14]. This comprises 4 statements, and consumers are asked to select which version of the text better reflects the statement. Consumers will also rate the texts according to two additional items asking which text " more clearly helps you know what to do next" and "would you be more likely to share with family and friends." (See survey attachment). Participants will receive a paper copy of these texts so that they are easier to compare side by side (see example of side-by-side text comparison), and can add comments to the paper copies to show which sections of the document are difficult to understand.

We expect it will take participants 15 minutes to complete the study.

Analysis

See analysis section for Part 1. This data will be used as a secondary outcome measure for the trial analyses.

Potential significance

This body of work delivers a novel, rigorously evaluated, co-designed, and policy-aligned digital tool to improve health literacy practices (the Health Literacy Editor).

References

[1] Australian Bureau of Statistics, Health literacy, Australia. Cat. No. 4233.0, Australian Bureau of Statistics, Canberra, Australia, 2006.

[2] N.D. Berkman, S.L. Sheridan, K.E. Donahue, D.J. Halpern, K. Crotty, Low health literacy and health outcomes: an updated systematic review, Ann Intern Med 155(2) (2011) 97-107. PMID:21768583.

[3] C. Cheng, M. Dunn, Health literacy and the Internet: a study on the readability of Australian online health information, Aust N Z J Public Health 39(4) (2015) 309-14. PMID:25716142.

[4] S.J. Shoemaker, M.S. Wolf, C. Brach, Development of the Patient Education Materials Assessment Tool (PEMAT): a new measure of understandability and actionability for print and audiovisual patient information, Patient Educ Couns 96(3) (2014) 395-403. PMID:24973195.

[5] A. Brega, J. Barnard, N. Mabachi, B. Weiss, D. DeWalt, C. Brach, M. Cifuentes, K. Albright, D. West, AHRQ Health Literacy Universal Precautions Toolkit, 2nd Edition, 2015. http://www.ahrq.gov/professionals/quality-patient-safety/quality-resources/tools/literacy-toolkit/healthlittoolkit2.html. (Accessed 14 June 2017.

[6] Office of Disease Prevention and Health Promotion, Health literacy online: A guide to simplifying the user experience, (2015).

[7] F. Mastroianni, Y.C. Chen, L. Vellar, E. Cvejic, J.K. Smith, K.J. McCaffery, D.M. Muscat, Implementation of an organisation-wide health literacy approach to improve the understandability and actionability of patient information and education materials: A pre-post effectiveness study, Patient Educ Couns 102(9) (2019) 1656-1661. PMID:30962076.

[8] G.H. McLaughlin, SMOG Grading-a New Readability Formula, Journal of Reading 12(8) (1969) 639-646.

[9] J.P. Kincaid, R.P. Fishburne Jr, R.L. Rogers, B.S. Chissom, Derivation of new readability formulas (automated readability index, fog count and flesch reading ease formula) for navy enlisted personnel, Naval Technical Training Command Millington TN Research Branch, 1975.

[10] L.W. Wang, M.J. Miller, M.R. Schmitt, F.K. Wen, Assessing readability formula differences with written health information materials: application, results, and recommendations, Res Social Adm Pharm 9(5) (2013) 503-16. PMID:22835706.

[11] K.L. Grabeel, J. Russomanno, S. Oelschlegel, E. Tester, R.E. Heidel, Computerized versus hand-scored health literacy tools: a comparison of Simple Measure of Gobbledygook (SMOG) and Flesch-Kincaid in printed patient education materials, J Med Libr Assoc 106(1) (2018) 38-45. PMID:29339932.

[12] O. Mac, J. Ayre, K. Bell, K. McCaffery, D.M. Muscat, Comparison of Readability Scores for Written Health Information Across Formulas Using Automated vs Manual Measures, JAMA Network Open 5(12) (2022) e2246051-e2246051.

[13] Clinical Excellence Commission, NSW Health Literacy Framework. 2019-2024, 2019. https://www.cec.health.nsw.gov.au/__data/assets/pdf_file/0008/487169/NSW-Health-Literacy-Framework-2019-2024.pdf. (Accessed 20 April 2022.

[14] L. Kelly, S. Ziebland, C. Jenkinson, Measuring the effects of online health information: Scale validation for the e-Health Impact Questionnaire, Patient Education and Counseling 98(11) (2015) 1418-1424.

[15] J. Brooke, Sus: a “quick and dirty’usability, Usability evaluation in industry 189(3) (1996).

[16] A. Bangor, P.T. Kortum, J.T. Miller, An empirical evaluation of the System Usability Scale, International Journal of Human-Computer Interaction 24(6) (2008) 574-594. PMID:WOS:000258821600003.

[17] F.D. Davis, Perceived Usefulness, Perceived Ease of Use, and User Acceptance of Information Technology, Mis Quarterly 13(3) (1989) 319-340. PMID:WOS:A1989CC00400006.
