# Supplementary material for: Online Plain Language Tool and Health Information Quality: A Randomized Clinical Trial
Source: JAMA Netw Open. 2024 Oct 8;7(10):e2437955. doi: 10.1001/jamanetworkopen.2024.37955 (PMC11581667; doi:10.1001/jamanetworkopen.2024.37955)
Supplement: Supplement 3. — Data Sharing Statement [file jamanetwopen-e2437955-s003.pdf]

Data Sharing Statement

Ayre. Online Plain Language Tool and Health Information Quality. *JAMA Netw Open*. Published October 08, 2024. doi:10.1001/jamanetworkopen.2024.37955

Data
Data available: No

Additional Information
Explanation for why data not available: Deidentified data will be made available on reasonable request
